# The vicinity of hyper-honeycomb *β*-Li_2_IrO_3_ to a three-dimensional Kitaev spin liquid state

**DOI:** 10.1038/srep29585

**Published:** 2016-07-12

**Authors:** Vamshi M. Katukuri, Ravi Yadav, Liviu Hozoi, Satoshi Nishimoto, Jeroen van den Brink

**Affiliations:** 1 Institute for Theoretical Solid State Physics, IFW Dresden, Helmholtzstr. 20, 01069 Dresden, Germany; 2 Institute for Theoretical Physics, Technische Universität Dresden, Helmholtzstr. 10, 01069 Dresden, Germany; 3 Department of Physics, Harvard University, Cambridge, Massachusetts 02138, USA

## Abstract

Due to the combination of a substantial spin-orbit coupling and correlation effects, iridium oxides hold a prominent place in the search for novel quantum states of matter, including, e.g., Kitaev spin liquids and topological Weyl states. We establish the promise of the very recently synthesized hyper-honeycomb iridate *β*-Li_2_IrO_3_ in this regard. A detailed theoretical analysis reveals the presence of large ferromagnetic first-neighbor Kitaev interactions, while a second-neighbor antiferromagnetic Heisenberg exchange drives the ground state from ferro to zigzag order via a three-dimensional Kitaev spin liquid and an incommensurate phase. Experiment puts the system in the latter regime but the Kitaev spin liquid is very close and reachable by a slight modification of the ratio between the second- and first-neighbor couplings, for instance via strain.

In magnetism, frustration refers to the existence of competing exchange interactions that cannot be simultaneously satisfied. Such effects can spawn new states of matter with quite exotic physical properties. Most famous in this regard are the different kinds of quantum spin liquids (QSL’s) that emerge from frustrated spin couplings^1^. In these collective states of matter quantum fluctuations are so strong that they disorder the spins even at the lowest temperatures. The types of QSL states that then emerge range from chiral ones^2^,^3^ to *Z*_2_ topological spin liquids^4^,^5^,^6^ carrying fractionalized excitations. Both experimentally and theoretically such QSL’s have been observed and intensely studied in two-dimensional (2D) systems^1^,^2^,^3^,^4^,^5^,^6^,^7^,^8^,^9^. How this situation carries over to three spatial dimensions (3D), in which tendencies towards formation of long-range ordered magnetic states are in principle stronger and the disordering effect of quantum fluctuations therefore less potent, is largely unexplored. This is not only due to the limitations of theoretical and numerical approaches in 3D but also to the the sparsity of relevant candidate materials^10^. Very recently the latter however fundamentally changed through the synthesis of insulating Li_2_IrO_3_ polymorphs^11^,^12^,^13^ in which the magnetic moments of Ir^4+^ ions form 3D honeycomb structures with threefold coordination. Here we concentrate on the *β*-Li_2_IrO_3_ polymorph, which forms a so-called hyper-honeycomb lattice, see Fig. 1. Such a lattice might in principle support a 3D Kitaev spin liquid^14^,^15^,^16^,^17^, a direct counterpart of its lower-dimensional, 2D equivalent^18^,^19^,^20^.

The 2D Kitaev-Heisenberg model on the honeycomb lattice is characterised by the presence of large uniaxial symmetric magnetic couplings that cyclically permute on the bonds of a given hexagonal ring^18^,^19^,^20^. A QSL phase is present in this model if the ratio between the Kitaev interaction *K* and Heisenberg coupling *J* is larger than 8^20^. Quasi-2D honeycomb compounds initially put forward for the experimental realization of the Kitaev-Heisenberg Hamiltonian are 5*d*^5^ and 4*d*^5^ *j* ≈ 1/2 systems^19^,^21^ such as Na_2_IrO_3_, *α*-Li_2_IrO_3_ and Li_2_RhO_3_. Subsequent measurements evidenced, however, either antiferromagnetically ordered^22^,^23^,^24^,^25^ or spin-glass^26^ ground states in these materials.

The three factors that complicate a straightforward materialisation of the Kitaev QSL ground state in the quasi-2D honeycomb compounds are the presence of (i) appreciable additional exchange anisotropies^27^,^28^,^29^, (ii) two crystallographically inequivalent Ir-Ir bonds and (iii) longer-range magnetic interactions between second- and third-neighbor iridium moments^22^,^23^,^27^,^30^,^31^. These additional interactions push quasi-2D Na_2_IrO_3_ and *α*-Li_2_IrO_3_ towards the formation of long-range antiferromagnetic (AF) order at temperatures below 15 K. Also the 3D honeycomb system *β*-Li_2_IrO_3_ orders magnetically: at 38 K the spins form an incommensurate (IC) ordering pattern^32^ with strong ferromagnetic (FM) correlations^11^. Apparently additional interactions beyond only the nearest-neighbor (NN) Kitaev and Heisenberg ones are relevant also in the 3D system. This leaves two main challenges: first, one would like to precisely quantify the different magnetic exchange interactions between the Ir moments and second, one should like to determine how far away the magnetic ground state is from a Kitaev-type 3D QSL. Here we meet these challenges through a combination of *ab initio* quantum chemistry calculations by which we determine the NN magnetic couplings in *β*-Li_2_IrO_3_ and exact diagonalization (ED) of the resulting effective spin Hamiltonian, on large clusters, to determine how far *β*-Li_2_IrO_3_ is situated from the QSL ground state in the magnetic phase diagram.

The *ab initio* results show that the NN exchange in *β*-Li_2_IrO_3_ is mostly FM, with relatively weak FM Heisenberg couplings of a few meV, large FM Kitaev interactions in the range of 10–15 meV, and additional anisotropies not included in the plain Kitaev-Heisenberg model. The sign and magnitude of second-neighbor Heisenberg couplings we determine from fits of the ED calculations to the experimental magnetization data. This second-neighbor effective coupling comes out as *J*_2_ ≈ 0.2–0.3 meV and is thus small and AF. Remarkably, this AF *J*_2_ stabilizes an IC magnetic structure that puts the system to be only a jot apart from the transition to a QSL ground state. Our findings provide strong theoretical motivation for further investigations on the material preparation side. The Kitaev QSL phase might be achieved by for instance epitaxial strain and relaxation in *β*-Li_2_IrO_3_ thin films, slightly modifying the *J*_2_/*K* ratio.

## Results

### Quantum Chemistry Calculations

Quantum chemistry calculations were first performed for the on-site *d*-*d* excitations, on embedded clusters consisting of one central octahedron and the three adjacent octahedra (for technical details, see Supplementary Information (SI) and ref. 33). Reference complete-active-space (CAS) multiconfigurational wave functions^34^ were in this case generated with an active orbital space defined by the five 5*d* functions at the central Ir site. While all possible occupations are allowed within the set of Ir 5*d* orbitals, double occupancy is imposed in the CAS calculations on the O 2*p* levels and other lower-energy orbitals. The self-consistent optimization was here carried out for an average of four states, i.e., 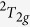 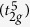 and the states of maximum spin multiplicity associated with each of the 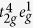 and 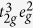 configurations. We then subsequently performed multireference configuration-interaction (MRCI) calculations^34^ with single and double excitations out of the Ir 5*d* and O 2*p* shells at the central octahedron. MRCI relative energies, without and with spin-orbit coupling (SOC), are listed in Table 1.

Due to slight distortion of the O cage^11^ and possibly anisotropic fields associated with the extended surroundings, the degeneracy of the Ir *t*_2*g*_ levels is lifted. Without SOC, the Ir 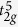 states are spread over an energy window of ≈0.1 eV (see Table 1). Similar results were earlier reported for the quasi-2D honeycomb iridates^33^. The low-symmetry fields additionaly remove the degeneracy of the *j* = 3/2 spin-orbit quartet. With orbitals optimized for an average of 5*d*^5^ states, i.e., 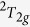 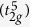, 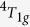 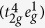, 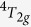 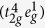 and 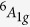 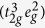, the *j* = 3/2-like components lie at 0.82 and 0.86 eV above the *j* ≈ 1/2 doublet, by MRCI + SOC computations (see Table 1). If the reference active space in the prior CAS self-consistent-field (CASSCF) calculation^34^ is restricted to only three (*t*_2*g*_) orbitals and five electrons, the relative energies of the *j* ≈ 3/2 components in the subsequent MRCI + SOC treatment are somewhat lower, 0.69 and 0.73 eV. The Ir *t*_2*g*_ to *e*_*g*_ transitions require excitation energies of at least 3 eV according to the MRCI data in Table 1, similar to values computed for *α*-Li_2_IrO_3_^33^.

While the quantum chemistry results for the on-site excitations in *β*-Li_2_IrO_3_ resemble very much the data for the quasi-2D honeycomb iridates, the computed intersite effective interactions show significant differences. The latter were estimated by MRCI + SOC calculations for embedded fragments having two edge-sharing IrO_6_ octahedra in the active region. As detailed in earlier work^27^,^35^,^36^, the *ab initio* quantum chemistry data for the lowest four spin-orbit states describing the magnetic spectrum of two NN octahedra is mapped in our scheme onto an effective spin Hamiltonian including both isotropic Heisenberg exchange and symmetric anisotropies. Yet the spin-orbit calculations, CASSCF or MRCI, incorporate all nine triplet and nine singlet states that arise from the two-Ir-site 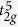
–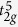 configuration (see SI). The MRCI treatment includes the Ir 5*d* electrons and the O 2*p* electrons at the two bridging ligand sites.

**Figure 1:**
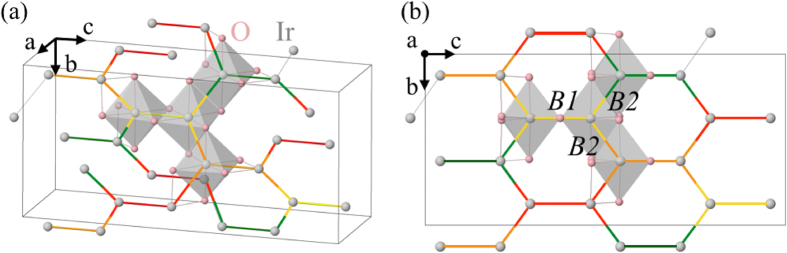
(**a**) Ir hyper-honeycomb lattice of *β*-Li_2_IrO_3_. The Ir-Ir links along the *c* axis, associated with equilateral Ir_2_O_2_ plaquettes^11^ and labeled *B1*, are shown in four different colors. *B1* links located at 0.125, 0.375, 0.625 and 0.875 on the *a* axis are shown in green, yellow, orange and red color, respectively. *B2* bonds connecting the *B1* links are shown in dual colors. O ions around four of the Ir sites are also shown. (**b**) Projection of the unit cell on the *bc* plane.

MRCI + SOC results for the NN effective couplings are listed in Table 2. The two, structurally different sets of Ir-Ir links are labeled *B1* and *B2*, see Fig. 1. For each of those, the O ions are distributed around the Ir sites such that the Ir-O-Ir bond angles deviate significantly from 90°. While the *B1* links display effective *D*_2_ point-group symmetry (the effective symmetry of a block of two NN octahedra is dictated not only by the precise arrangement of the O ions coordinating the two magnetically active Ir sites but also by the symmetry of the extended surroundings), the *B2* bonds possess *C*_*i*_ symmetry, slightly away from *C*_2*h*_ due to small differences between the Ir-O bond lengths on the Ir_2_O_2_ plaquette of two Ir ions and two bridging ligands (2.025 vs 2.023 Å^11^). The absence of an inversion center allows a nonzero antisymmetric exchange on the *B1* links. However, our analysis shows this antisymmetric Dzyaloshinskii-Moriya coupling is the smallest effective parameter in the problem — two orders of magnitude smaller than the dominant NN interactions, i.e., the Kitaev exchange. On this basis and further symmetry considerations (see the discussion in refs 27, 35, 36, 37), the effective spin Hamiltonian for the *B1* links is assumed *D*_2*h*_-like and in the local Kitaev reference frame (with the *z* axis perpendicular to the Ir_2_O_2_ plaquette and *x, y* within the plane of the plaquette^19^,^27^) it reads


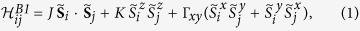


where 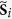 and 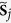 are pseudospin 1/2 operators, *K* defines the Kitaev component and Γ_*xy*_ is the only non-zero off-diagonal coupling of the symmetric anisotropic tensor.

For the *B2* units of edge-sharing IrO_6_ octahedra, the effective spin Hamiltonian reads in the local Kitaev coordinate frame as


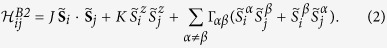


We find for the *B2* links that slight distortions lowering the bond symmetry from *C*_2*h*_ to *C*_*i*_ have minor effects on the computed wave functions and the quantum chemistry data can be safely mapped onto a *C*_2*h*_ model. For *C*_2*h*_ symmetry, the elements of the symmetric anisotropic tensor are such that Γ_*zx*_ = −Γ_*yz*_.

The wave functions for the low-lying four states in the two-Ir-site problem can be conveniently expressed in terms of 1/2 pseudospins as in Table 2. In *D*_2_ symmetry (*B1* links) these pseudospin wave functions, singlet Φ_S_ and triplet Φ_1_, Φ_2_, Φ_3_, transform according to the *A*_*u*_, *B*_2_, *B*_1_ and *A*_*u*_ irreducible representations, respectively. For (nearly) *C*_2*h*_ symmetry (*B2* links), Φ_S_, Φ_1_, Φ_2_ and Φ_3_ transform according to *A*_*g*_, *B*_*u*_, *B*_*u*_ and *A*_*u*_, respectively. The amount of Φ_S_–Φ_3_ (*B1*) and Φ_1_–Φ_2_ (*B2*) mixing (see Table 2) is determined by analysis of the “full” spin-orbit wave functions obtained in the quantum chemistry calculations.

As seen in Table 2, for each set of Ir-Ir links in *β*-Li_2_IrO_3_, *B1* and *B2*, both *J* and *K* are FM. In contrast, *J* is AF for all pairs of Ir NN’s in honeycomb Na_2_IrO_3_^27^ and features different signs for the two types of Ir-Ir links in *α*-Li_2_IrO_3_^35^. The Kitaev exchange, on the other hand, is found to be large and FM in all 213 compounds, see Table 2 and refs 27 and 35. In addition to the Kitaev coupling, sizable off-diagonal symmetric anisotropic interactions are predicted. In *β*-Li_2_IrO_3_, these are FM for the *B1* bonds and show up with both + and − signs for the *B2* links (the sign of these terms is with respect to the local Kitaev reference frame), see Table 2.

### Magnetic Phase Diagram

**Figure 2:**
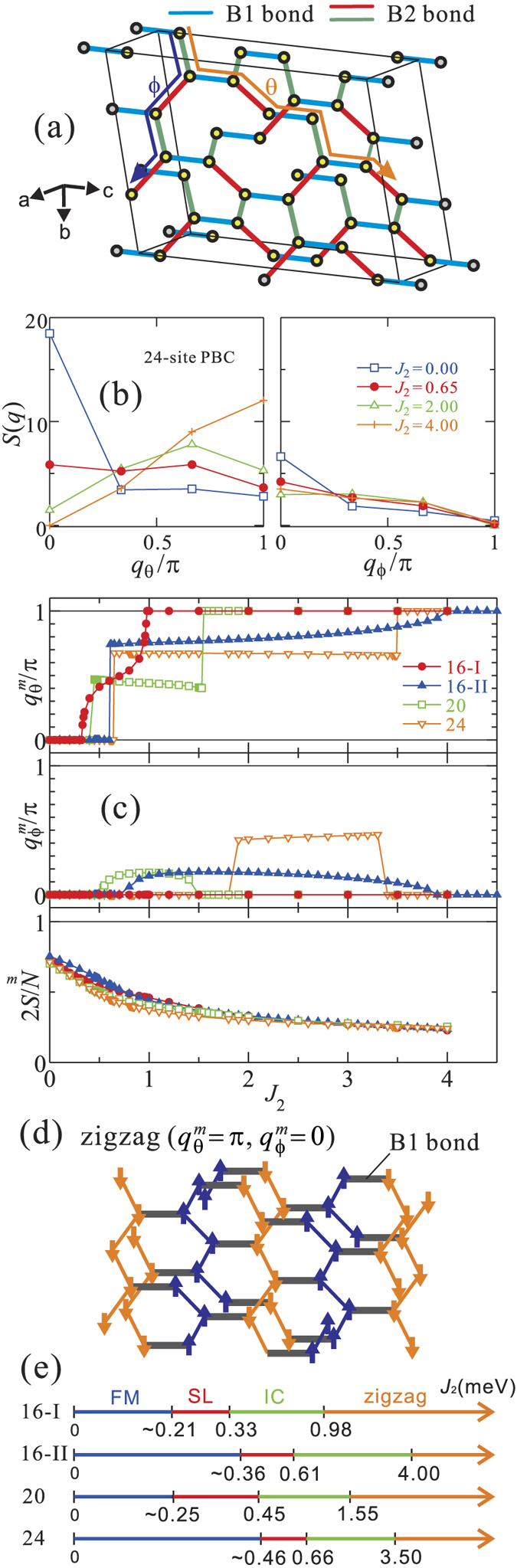
(**a**) Sketch of the 24-site “periodic” cluster. (**b**) Static spin-structure factor along paths *θ* and *ϕ*, see text. (**c**) Propagation vectors 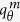, 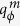 and total spin 2 *S*/*N* for the periodic clusters, as functions of *J*_2_. (**d**) AF zigzag order on the hyper-honeycomb lattice. (**e**) Magnetic phase diagrams obtained for the periodic clusters.

Having established the nature and the magnitude of the NN effective spin couplings, we now turn to the magnetic phase diagram of *β*-Li_2_IrO_3_. In addition to the NN MRCI + SOC data of Table 2, we have to take into account explicitly the second-neighbor Heisenberg interactions. Due to the 3D nature of the iridium lattice, with alternate rotation of two adjacent *B2* bonds around the *B1* link with which both share an Ir ion, one can safely assume that the third-neighbor exchange is vanishingly small. Results of ED calculations for an extended (pseudo)spin Hamiltonian including the MRCI NN interactions and a variable second-neighbor Heisenberg coupling parameter *J*_2_ are shown in Fig. 2. Different types of clusters were considered, with either 16, 20 or 24 Ir sites. The 24-site cluster used in ED calculations with periodic boundary conditions is displayed in Fig. 2(a) while the structure of the smaller clusters is detailed in SI.

In order to investigate the magnetic properties of *β*-Li_2_IrO_3_, we calculated the static spin-structure factor 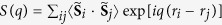 along two paths denoted as *θ* (*bc*-diagonal) and *ϕ* (*ab*-diagonal) in Fig. 2(a), where the distance between neighboring *B1* bonds is taken as 1. The results for several *J*_2_ values with the 24-site cluster are plotted in Fig. 2(b). The propagation vector for each path 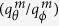, determined as the wave number *q* providing a maximum of *S*(*q*), is plotted in Fig. 2(c). For *J*_2_ = 0 the ground state is characterized by long-range FM order, i.e., 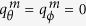, consistent with a previous classical Monte Carlo study^38^,^39^. Given the strong FM character of the NN exchange, ground states different from FM order are only obtained for finite AF *J*_2_. With increasing strength of the AF *J*_2_, *q*_*θ*_ develops finite values starting at *J*_2_ = *J*_2,*c*1_ and reaches *π* at *J*_2_ = *J*_2,*c*2_ whereas *q*_*ϕ*_ is finite but small in the range *J*_2,*c*1_ < *J*_2_ < *J*_2,*c*2_ and zero otherwise. This evidences two magnetic phase transitions, from FM to IC order and further to a commensurate ground state. The latter commensurate structure corresponds to zigzag AF order, a schematic picture of which is shown in Fig. 2(d). The ED results for the four different types of periodic clusters are here in good overall agreement, as shown in Fig. 2(e). Some differences arise only with respect to the precise position of the critical points.

An intriguing feature is the appearance of a SL state in between the FM and IC phases. Since the total spin 2 *S*/*N* falls off rapidly and continuously near *J*_2_ = *J*_2,*c*1_ [see Fig. 2(c)], the FM ground state is expected to change into SL before reaching the IC regime. It can be confirmed by a structureless static spin-structure factor, like nearly flat *q*-dependence of *S*(*q*) at *J*_2_ = 0.65 in Fig. 2(b). In Fig. 2(e) we also provide the critical values marking the transition between the FM and SL states. This was estimated as the point where any of the 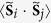 expectation values turn negative, which implies a collapse of long-range FM order. Importantly, we find that the SL phase shows up in each of the four different types of periodic clusters. A more detailed analysis of the spin-spin correlations is provided in SI.

## Discussion

**Figure 3:**
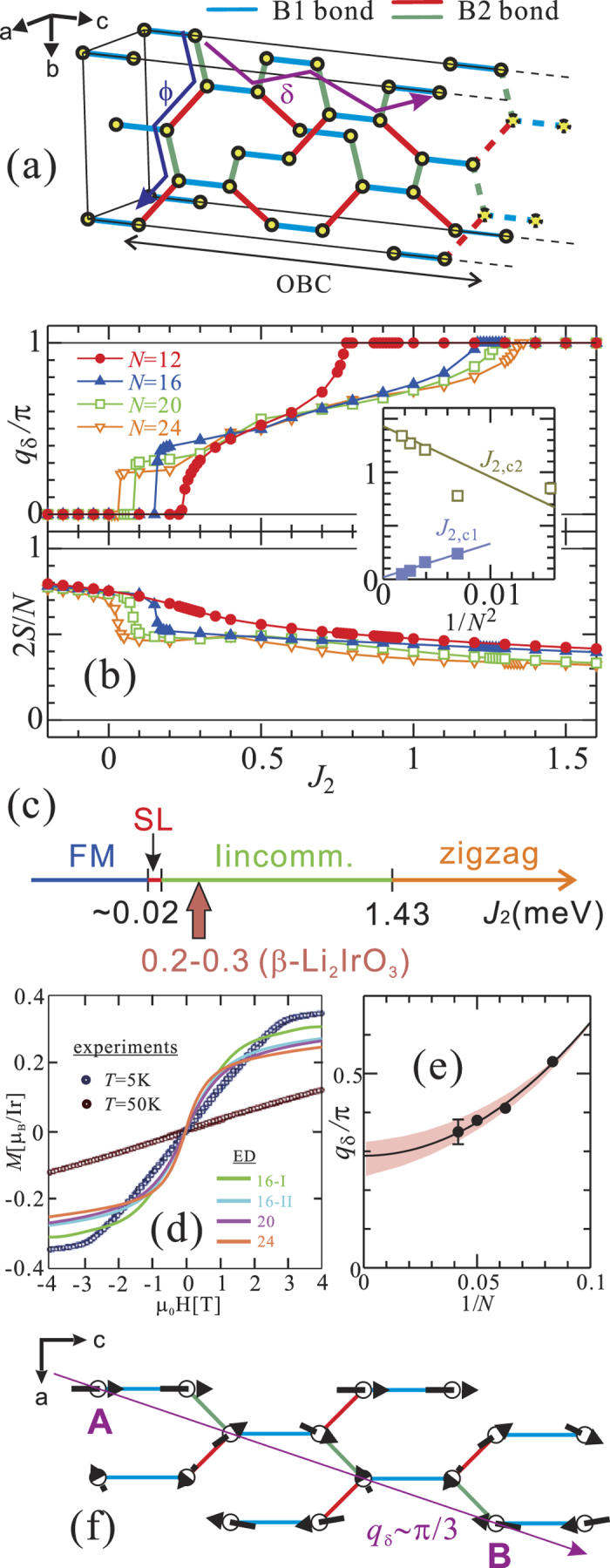
(**a**) Sketch of the cluster with open boundaries along the *c* direction. (**b**) Propagation vector 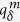 and total spin 2 *S*/*N* for our “open” clusters, as function of *J*_2_. Inset: finite-scaling analysis of the critical points. (**c**) Magnetic phase diagrams obtained by ED. (**d**) Experimental (see ref. 11) and theoretical magnetization curves for *β*-Li_2_IrO_3_. The latter are obtained with either *J*_2_ = 0.2 (periodic 16- and 20-site clusters) or *J*_2_ = 0.3 meV (periodic 24-site cluster) and the NN MRCI couplings from Table 2. (**e**) Finite-scaling analysis of 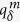 at *J*_2_ = 0.3 meV using the open clusters. (**f**) Experimental results of the magnetic structure for *β*-Li_2_IrO_3_ (see ref. 32).

Typically, a commensurate-to-IC transition critical point tends to be overestimated by using periodicity. For estimating more precisely the critical *J*_2_ values we therefore additionally studied clusters with open boundary conditions along the *c* direction. Also, for a direct comparison between our ED results and the experimentally observed magnetic structure, we introduce an additional path *δ* (*ac*-diagonal), sketched in Fig. 3(a). The size of the cluster along *a* and *b* has insignificant effect on the computed critical *J*_2_ values because 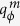 (*ab*-diagonal) is either zero, around the critical points (periodic 24-site cluster), or very small, in the IC phase (periodic 16- and 20-site clusters), as seen in Fig. 2(c).

The value of the propagation vector along the *δ*-path 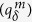 is shown in Fig. 3(b) as function of *J*_2_ for various cluster “lengths” in the *c* direction. The inset displays a finite-size scaling analysis for the critical values. In the infinite-length limit, we find *J*_2,*c*1_ = 0.02 and *J*_2,*c*2_ = 1.43 meV. The corresponding phase diagram is provided in Fig. 3(c). Similar critical points, i.e., *J*_2,*c*1_ = 0.02 and *J*_2,*c*2_ = 1.48 meV, are obtained for 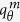 (see SI).

As shown in Fig. 3(b), the dropdown of 2 *S*/*N* near *J*_2_ = *J*_2,*c*1_ is more clearly seen than in the case of periodic clusters because the formation of IC order is not hindereded for open clusters. Defining the FM-SL *J*_2,*c*1_ critical value as the point where 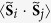 turns negative for any (*i, j*) pair, the SL phase in the vicinity of *J*_2_ ≈ *J*_2,*c*1_ = 0.02 meV would have a width of about 0.01 *J*_2_. In other words, a very tiny FM *J*_2_ coupling may drive the system from FM order to a SL state. With further increasing *J*_2_, the system goes through an IC phase to AF zigzag order at *J*_2_ = 1.43 meV.

To finally determine the value of *J*_2_ in *β*-Li_2_IrO_3_, we fitted the magnetization curve obtained by ED calculations at *T* = 0 K [see Fig. 3(d)] to the experimental data at *T* = 5 K^11^. Such an exercise yields *J*_2_ = 0.2–0.3 meV, i.e., *J*_2_ ≈ 0.1 *J*_2,*c*2_, so that the system is relatively far from the instability to zigzag order but very close to the transition to the SL ground state. Since with increasing *J*_2_ the propagation vector 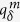 of the IC phase increases smoothly from that of the SL 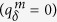 to that of the zigzag state 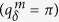, long-wavelength IC order with a small propagation vector is expected for *β*-Li_2_IrO_3_. By performing a finite-size scaling analysis of 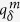 at *J*_2_ = *J*_2,*c*1_(*N*) + 0.28 meV, we obtain 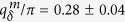 for *J*_2_ = 0.3 meV in the infinite-length limit. An experiment-based estimate for 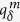 can be extracted from recent magnetic resonant x-ray diffraction data^32^ [see Fig. 3(f)]; the spins on sites A and B (their distance is three lattice spacings) have almost opposite directions, which leads to 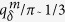. That fits reasonably well our theoretical estimate. The stabilization of an IC state by *J*_2_ couplings has been previously discussed for 1D zigzag chains like the path we label here as *δ* in ref. 40.

The value extracted for *J*_2_ from our fit of the magnetization data is thus within our theoretical framework fully consistent with the experimentally observed IC magnetic order in *β*-Li_2_IrO_3_. Nevertheless we find that the system is remarkably close to a three-dimensional spin-liquid ground state, which can be reached by a minute change of ~0.25 meV, an energy scale that corresponds to about 3 K, in the second-neighbour exchange parameter *J*_2_. Changes of this order of magnitude can easily be induced by pressure or strain.

## Additional Information

**How to cite this article**: Katukuri, V. M. *et al*. The vicinity of hyper-honeycomb *β*-Li_2_IrO_3_ to a three-dimensional Kitaev spin liquid state. *Sci. Rep.* **6**, 29585; doi: 10.1038/srep29585 (2016).

## Supplementary Material

Supplementary Information

## Figures and Tables

**Table 1:** Ir^4+^ 5*d* ^5^ multiplet structure in *β*-Li_2_IrO_3_, all numbers in eV.

States	MRCI	MRCI + SOC (×2)
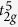	0, 0.07, 0.11 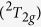	0 (*j* ≈ 1/2)
		0.82, 0.86 (*j* ≈ 3/2)
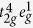	2.99, 3.01, 3.02 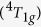	3.32, … 3.79
	3.60, 3.65, 3.66 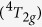	4.23, … 4.50
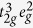	5.01 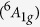	5.87, … 5.87

Due to the noncubic environment, the *T*_2*g*_/*T*_1*g*_ (and spin-orbit coupled *j* = 3/2) states are split appart. We still use however notations corresponding to *O*_*h*_ symmetry. Only the lowest and highest Kramers doublets are shown for each set of higher-lying spin-orbit states.

**Table 2:** MRCI splittings among the four low-lying magnetic states and effective exchange couplings (meV) for two NN IrO_6_ octahedra in *β*-Li_2_IrO_3_.

Energies & effective couplings	*B1*^1^	*B2*^2^
*E*_2_ (Ψ_2_)	0.0	0.0
*E*_3_ (Ψ_3_)	2.1	4.2
*E*_1_ (Ψ_1_)	8.4	8.3
*E*_S_ (Ψ_S_)	8.7	10.5
*J*	−0.3	−2.4
*K*	−14.7	−11.7
Γ_*xy*_	−2.1	−3.9
Γ_*zx*_ = −Γ_*yz*_	—	2.0

A *local* coordinate frame is used for each Ir-Ir link (*x* along the Ir-Ir bond, *z* perpendicular to the Ir_2_O_2_ plaquette). For *B1* bonds, the weight of Φ_S_ in Ψ_S_ and of Φ_3_ in Ψ_3_ is ≈99%. For *B2* links, the Φ_1_–Φ_2_ mixing is approximately 3–97%, where 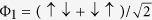, 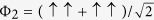, 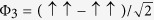 and 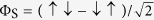, see text.

^1^
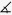
(Ir-O-Ir) = 94.7°, *d*(Ir-Ir) = 2.98, *d*(Ir-O_1,2_) = 2.025 Å^11^.

^2^
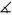
(Ir-O-Ir) = 94.4°, *d*(Ir-Ir) = 2.97, *d*(Ir-O_1_) = 2.025, *d*(Ir-O_2_) = 2.023 Å^11^. O_1_ and O_2_ are the two bridging O’s.
