# Supplementary material for: The vicinity of hyper-honeycomb β-Li2IrO3 to a three-dimensional Kitaev spin liquid state
Source: Sci Rep. 2016 Jul 12;6:29585. doi: 10.1038/srep29585 (PMC4941717; doi:10.1038/srep29585)
Supplement: Supplementary Information [file srep29585-s1.pdf]

Supplementary Information — The vicinity of hyper-honeycomb β-Li_2IrO_3 to a three-dimensional Kitaev spin liquid state

Vamshi M. Katukuri,[1] Ravi Yadav,[1] Liviu Hozoi,[1] Satoshi Nishimoto,[1,2] and Jeroen van den Brink[1,3]

[1]*Institute for Theoretical Solid State Physics, IFW Dresden, Helmholtzstrasse 20, 01069 Dresden, Germany*
[2]*Institute for Theoretical Physics, Technische Universität Dresden, Helmholtzstrasse 10, 01069 Dresden, Germany*
[3]*Department of Physics, Harvard University, Cambridge, Massachusetts 02138, USA*

(Dated: March 16, 2016)

I. QUANTUM CHEMISTRY CALCULATIONS

Ir d-shell excitations. On-site d-d excitations for Ir ions in β-Li_2IrO_3 were obtained from *ab initio* quantum chemistry (QC) calculations performed on a cluster consisting of one reference IrO_6 octahedron plus three nearest-neighbor (NN) IrO_6 octahedra and 15 nearby Li ions. The surrounding solid-state matrix was modeled as a finite array of point charges fitted to reproduce the crystal Madelung field in the cluster region. We used energy-consistent relativistic pseudopotentials for Ir, with quadruple-zeta basis sets for the valence shells of the reference Ir ion along with two f polarization functions and triple-zeta basis functions for the Ir NN's[1]. The oxygen ligands of the central octahedron were represented by all-electron triple-zeta basis sets[2]. For the Li^+ NN's we employed total-ion effective potentials and a single s valence basis function[3]. To simplify the analysis of the spin-orbit coupled wave functions, the NN Ir^{4+} ions were modeled as closed-shell Pt^{4+} t_{2g}^6 species.[4–6]

At the complete-active-space self-consistent-field (CASSCF) level, two different sets of calculations were performed, with different active orbital spaces. In a first set of computations, all five $5d$ functions ($t_{2g}+e_g$) at the central Ir site and five electrons were considered as active; the orbitals were optimized for an average of the $^2T_{2g}$ (t_{2g}^5), $^4T_{1g}$ ($t_{2g}^4e_g^1$), $^4T_{2g}$ ($t_{2g}^4e_g^1$), and $^6A_{1g}$ ($t_{2g}^3e_g^2$) states. All these states were included in the subsequent spin-orbit treatment. In the second set of calculations, the active space was truncated to contain only the $5d$ t_{2g} orbitals of the central Ir site and five electrons. The orbitals were here optimized for the $^2T_{2g}$ (t_{2g}^5) configuration and the off-diagonal spin-orbit couplings between the $^2T_{2g}$ (t_{2g}^5) and $t_{2g}^4e_g^1$ states were neglected. In both cases, all O $2p$ and Ir t_{2g} electrons at the central octahedron were correlated in the subsequent multireference configuration interaction (MRCI) calculations. The QC pacakge MOLPRO[7] was employed for all computations.

NN magnetic couplings. The magnetic spectrum of two NN Ir^{4+} ions was obtained from CASSCF and MRCI spin-orbit calculations on units of two edge-sharing IrO_6 octahedra. To accurately describe the charge distribution at sites in the immediate neighborhood, we also included in the actual cluster the closest 22 Li^+ ions and the four adjacent IrO_6 octahedra around the reference [Ir_2O_{10}] fragment. As for the single-site calculations, we used energy-consistent relativistic pseudopotentials with quadruple-zeta basis sets for the valence shells of the two reference Ir ions[1], all-electron quintuple-zeta basis sets for the two bridging ligands[2] and triple-zeta basis functions for the other O's of the two reference octahedra[2]. Additionally, we employed two Ir f[1] and four O d polarization functions for the two central Ir ions and the two bridging ligands,[2] respectively. The NN Ir^{4+} sites were once again modeled as closed-shell Pt^{4+} t_{2g}^6 species.

Multiconfiguration reference wave functions were first generated by CASSCF calculations. For two NN IrO_6 octahedra, the finite set of Slater determinants was defined in the CASSCF treatment in terms of ten electrons (2×5) and six Ir t_{2g} orbitals (2×3). The SCF optimization was carried out for an average of the lowest nine singlet and the nine triplet states associated with this manifold. All these states entered the spin-orbit calculations, both at the CASSCF and MRCI levels. On top of the CASSCF reference, the MRCI expansion additionally includes single and double excitations from the Ir t_{2g} shells and the $2p$ orbitals of the bridging ligands. A similar strategy of explicitly dealing only with selected groups of ligand orbitals was earlier adopted in QC studies on both $3d$[8–11] and $5d$[4–6,12,13] compounds, with results in good agreement with the experiment[4,6,8–12]. To separate the Ir $5d$ and O $2p$ valence orbitals into different groups, we used the Pipek-Mezey[14] orbital localization module available in MOLPRO[7].

II. EFFECTIVE SPIN HAMILTONIAN

For a pair of NN pseudospins $\tilde{\mathbf{S}}_i$ and $\tilde{\mathbf{S}}_j$, the most general bilinear spin Hamiltonian can be written as

$$\mathcal{H}_{ij} = J_{ij}\,\tilde{\mathbf{S}}_i\cdot\tilde{\mathbf{S}}_j + \mathbf{D}_{ij}\cdot\tilde{\mathbf{S}}_i\times\tilde{\mathbf{S}}_j + \tilde{\mathbf{S}}_i\cdot\mathbf{\Gamma}_{ij}\cdot\tilde{\mathbf{S}}_j\,, \tag{1}$$

where J_{ij} is the isotropic Heisenberg exchange, the vector $\mathbf{D}_{ij}$ defines the antisymmetric anisotropy, and $\mathbf{\Gamma}_{ij}$ is a symmetric traceless second-rank tensor that describes the symmetric part of the exchange anisotropy.

***B1* links.** The Ir hyper-honeycomb lattice contains two structurally different sets of Ir-Ir links,[15] labeled as *B1* and *B2* in Fig. 1 of the main text. For the *B1* links, the magnetic spectrum was mapped onto an effective spin Hamiltonian displaying D_{2h}

point-group symmetry. In the reference frame $\{\mathbf{X},\mathbf{Y},\mathbf{Z}\}$ with the Ir-Ir bond along the ($\mathbf{X}$) axis and $\mathbf{Z}$ perpendicular to the Ir_2O_2 plaquette, see Fig. 1a, the effective magnetic Hamiltonian reads

$$\mathcal{H}^{B1}_{\langle ij\rangle} = J^{(0)}\tilde{\mathbf{S}}_i\cdot\tilde{\mathbf{S}}_i + A\,(\tilde{S}^X_i\tilde{S}^X_j - \tilde{S}^Z_i\tilde{S}^Z_j) + B\,(\tilde{S}^Y_i\tilde{S}^Y_i - \tilde{S}^Z_i\tilde{S}^Z_j). \tag{2}$$

The antisymmetric anisotropy is the smallest parameter according to our analysis and therefore neglected in the following. Further, only the diagonal elements of the symmetric anisotropic tensor may have, by symmetry, nonzero values. Diagonalization of (2) yields the eigenvalues $E_{\rm S} = \frac{3J^{(0)}}{4}$, $E_1 = \frac{J^{(0)}+2A+2B}{4}$, $E_2 = \frac{J^{(0)}-2B}{4}$ and $E_3 = \frac{J^{(0)}-2A}{4}$. The effective exchange couplings are then given by

$$\begin{aligned} J^{(0)} &= \frac{1}{3}(E_1+E_2+E_3) - E_S, \\ A &= \frac{2}{3}(E_1+E_2-2E_3), \\ B &= \frac{2}{3}(E_1-2E_2+E_3). \end{aligned} \tag{3}$$

In the local Kitaev reference system $\{\mathbf{x},\mathbf{y},\mathbf{z}\}$, that is rotated from the reference frame $\{\mathbf{X},\mathbf{Y},\mathbf{Z}\}$ by 45° about the $\mathbf{Z}=\mathbf{z}$ axis (see Fig.1a and Refs. 5,16), the Hamiltonian given in expression (2) above is transformed into equation (1) of the main text. For the latter, the exchange interaction parameters read

$$J = J^{(0)} + \frac{A+B}{2}\ ,\quad K = -\frac{3}{2}(A+B)\ ,\quad \Gamma_{xy} = \frac{A-B}{2} \equiv D. \tag{4}$$

B2 **links.** The magnetic spectrum obtained for the Ir-Ir links of type *B2* was mapped onto an effective spin Hamiltonian displaying C_{2h} point-group symmetry[15]:

$$\mathcal{H}^{B2}_{\langle ij\rangle} = \tilde{\mathbf{S}}_i\cdot\begin{pmatrix} J^{(0)}+A & 0 & 0 \\ 0 & J^{(0)}+B & C \\ 0 & C & J^{(0)}-(A+B)\end{pmatrix}\cdot\tilde{\mathbf{S}}_j\,. \tag{5}$$

A straightforward diagonalization of $\mathcal{H}_{\langle ij\rangle}$ in (5) yields the following eigenvalues and eigenfunctions:

$$\begin{aligned} E_{\rm S} &= -\frac{3J^{(0)}}{4}\,, & \Phi_{\rm S} &= \frac{\uparrow\downarrow-\downarrow\uparrow}{\sqrt{2}}, \\ E_1 &= \frac{J^{(0)}+A+\sqrt{(A+2B)^2+4C^2}}{4}\,, & \Phi_1 &= \frac{\uparrow\downarrow+\downarrow\uparrow}{\sqrt{2}}, \\ E_2 &= \frac{J^{(0)}+A-\sqrt{(A+2B)^2+4C^2}}{4}\,, & \Phi_2 &= \frac{\uparrow\uparrow+\downarrow\downarrow}{\sqrt{2}}, \\ E_3 &= \frac{J^{(0)}-2A}{4}\,, & \Phi_3 &= \frac{\uparrow\uparrow-\downarrow\downarrow}{\sqrt{2}}. \end{aligned} \tag{6}$$

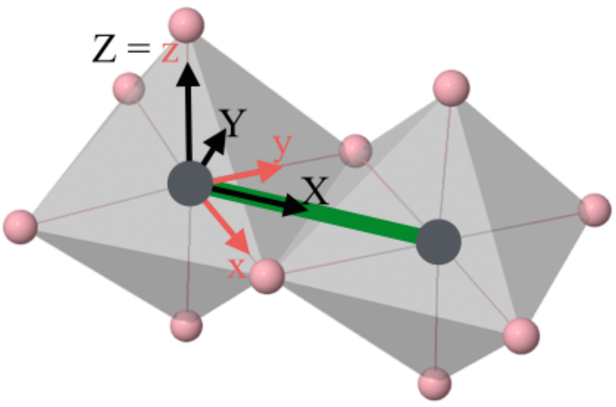

FIG. 1: Local reference frames $\{\mathbf{X},\mathbf{Y},\mathbf{Z}\}$ and $\{\mathbf{x},\mathbf{y},\mathbf{z}\}$ for a Ir_2O_{10} block of two NN octahedra.

Here $\Phi_{\rm S}$ is the total spin singlet and Φ_{1-3} describe the three triplet components. The diagonalization procedure to obtain the expressions in (6) is equivalent with a rotation of the coordinate system $\{\mathbf{X},\mathbf{Y},\mathbf{Z}\}$ around $\mathbf{X}$ by an angle α to a new frame $\{\mathbf{X}',\mathbf{Y}',\mathbf{Z}'\}$ in which the symmetric anisotropic exchange matrix is diagonal.[17] $\{\mathbf{X}',\mathbf{Y}',\mathbf{Z}'\}$ are also referred to as principal axes and the angle α is given by

$$\tan(2\alpha) = \frac{2C}{A+2B}\,. \tag{7}$$

In C_{2h} symmetry, the $\Phi_{\rm S}$, Φ_1, Φ_2 and Φ_3 spin-orbit wave functions transform according to the A_g, B_u, B_u and A_u irreducible representations, respectively. Since states Φ_1 and Φ_2 belong to the same irreducible representation B_u, they are in general admixed, i.e., in the reference frame $\{\mathbf{X},\mathbf{Y},\mathbf{Z}\}$ the corresponding eigenfunctions should be written as

$$\begin{aligned}\Psi_1 &= \Phi_1\cos\alpha + i\Phi_2\sin\alpha\,,\\ \Psi_2 &= i\Phi_1\sin\alpha + \Phi_2\cos\alpha\,.\end{aligned} \tag{8}$$

The mixing parameter $\xi = \sin\alpha$ reads

$$i\xi = \langle\Phi_2|\Psi_1\rangle = \langle\Phi_1|\Psi_2\rangle \tag{9}$$

and is explicitly obtained from the QC data.

Since the QC calculations were actually performed in C_1 symmetry, to determine the nature of each of the lowest four spin-orbit states, we explicitly computed the dipole and quadrupole transition matrix elements within that manifold. Standard selection rules and the nonzero dipole and quadrupole matrix elements in the QC outputs then clearly indicate which state is which. We also carried out the transformation of the spin-orbit wave functions from the usual $\{L_1,M_{L_1},L_2,M_{L_2},S,M_S\}$ basis in standard QC programs to the $\{\tilde{S}_1,\tilde{S}_2,\tilde{M}_{S_1},\tilde{M}_{S_2}\}$ basis. This allows the study of Φ_1–Φ_2 mixing due to the off-diagonal Γ_{yz} and Γ_{zx} couplings. With such an analysis, we find that in β-Li_2IrO_3 the weight of Φ_1 in Ψ_1 (and of Φ_2 in Ψ_2) is 97% for links *B2*.

Using Eqs. (6), (7) and (9) we obtain the effective coupling parameters of (5) as:

$$\begin{aligned}J^{(0)} &= \frac{1}{3}(E_1+E_2+E_3) - E_S,\\ A &= \frac{2}{3}(E_1+E_2) - \frac{4}{3}E_3,\\ B &= \frac{1}{2}\left[-A \pm \frac{2(E_1-E_2)}{\sqrt{1+\eta^2}}\right],\ \text{with}\ \eta = \frac{2\xi\sqrt{1-\xi^2}}{1-2\xi^2},\\ C &= \frac{\eta(A+2B)}{2}\,.\end{aligned} \tag{10}$$

In the local Kitaev reference system $\{\mathbf{x},\mathbf{y},\mathbf{z}\}$, the Hamiltonian given in expression (5) above is transformed into equation (2) of the main text and the exchange interaction parameters become

$$J = J^{(0)} + \frac{A+B}{2}\,,\ \ K = -\frac{3}{2}(A+B)\,,\ \ \Gamma_{xy} = \frac{A-B}{2} \equiv D\,,\ \ \Gamma_{zx} = \frac{-C}{\sqrt{2}}\,,\ \ \Gamma_{yz} = \frac{C}{\sqrt{2}}\,. \tag{11}$$

The numerical values of the above coupling parameters, as found by *ab initio* MRCI calculations, are (in units of meV) :

Bond type b	$J_b^{(0)}$	A_b	B_b	C_b	$J_b = J_b^{(0)} + \frac{A_b+B_b}{2}$	$K_b = -\frac{3}{2}(A_b+B_b)$	$\Gamma^b_{xy} = \frac{A-B}{2}$	$\Gamma^b_{yz} = -\Gamma^b_{zx}$
1($B1$)	−5.2	2.8	7.0	–	−0.3	−14.7	−2.1	–
2($B2$)	−6.33	−0.07	7.84	2.83	−2.45	−11.65	−3.95	2.0
3($B2$)	−6.33	−0.07	7.84	2.83	−2.45	−11.65	−3.95	2.0

For completeness, we also provide the coupling parameters as found by spin-orbit CASSCF calculations (in units of meV) :

Bond type b	$J_b^{(0)}$	A_b	B_b	C_b	$J_b = J_b^{(0)} + \frac{A_b+B_b}{2}$	$K_b = -\frac{3}{2}(A_b+B_b)$	$\Gamma^b_{xy} = \frac{A_b-B_b}{2}$	$\Gamma^b_{yz} = -\Gamma^b_{zx}$
1($B1$)	−4.77	2.87	3.87	–	−1.40	−10.11	−0.50	–
2($B2$)	−5.93	0.53	4.62	1.77	−3.36	−7.73	2.04	1.25
3($B2$)	−5.93	0.53	4.62	1.77	−3.36	−7.73	2.04	1.25

III. CLUSTERS USED FOR THE EXACT-DIAGONALIZATION CALCULATIONS

The structural building block of the Ir hyper-honeycomb lattice includes four Ir sites and is shown in Fig. 2(a). For the exact-diagonalization (ED) calculations with periodic boundary conditions (PBC) we used four different types of clusters: two 16-site clusters [see Fig. 2(b–c)], a 20-site cluster [Fig. 2(d)] and a 24-site cluster [Fig. 2(a) in the main text].

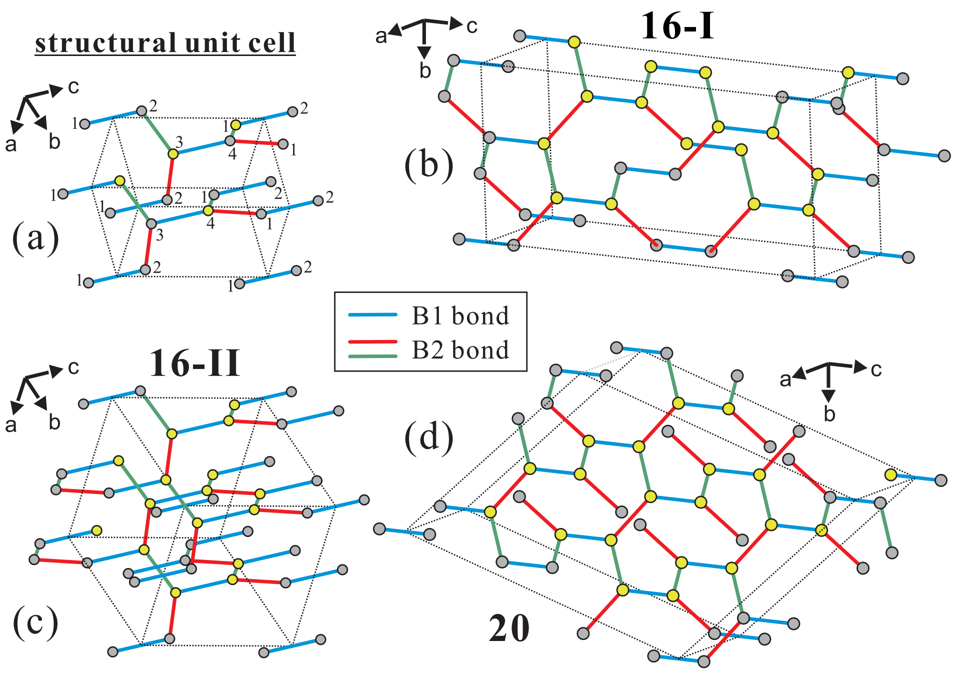

FIG. 2: (a) Structural building block for the Ir hyper-honeycomb lattice of β-Li_2IrO_3. (b–c) 16-site and (d) 20-site clusters used for ED calculations with periodic boundary conditions, denoted as 16-I, 16-II and 20, respectively.

IV. FITTING OF THE MAGNETIZATION CURVE

In Fig. 3, magnetization curves are plotted as functions of the magnetic field for several J_2 values. Here, the 16-I cluster has been used. The magnetization curves are quite sensitive to the J_2 value and enable us to perform a very fine fitting of the experimental data.

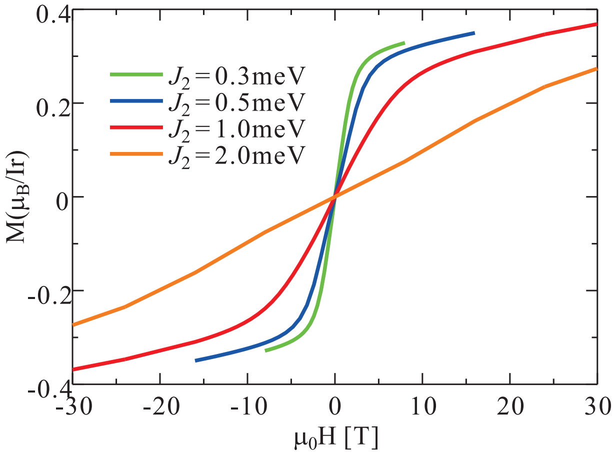

FIG. 3: Magnetization curves calculated by ED for the 16-I cluster.

V. SPIN-SPIN CORRELATIONS IN THE SPIN LIQUID PHASE

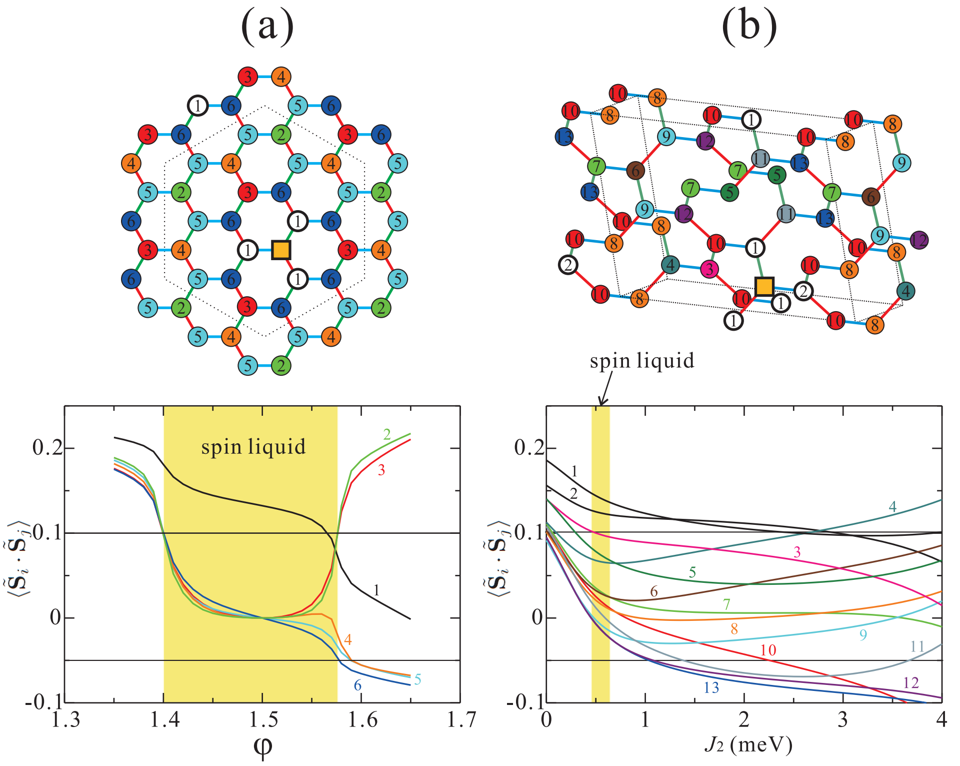

FIG. 4: Spin-spin correlation functions $\langle \tilde{\mathbf{S}}_i \tilde{\mathbf{S}}_j \rangle$ and sketch of the "periodic" clusters used in the ED calculations for (a) a 2D Kitaev-Heisenberg and (b) our hyper-honeycomb models. The reference site is indicated by a square and the numbers labelling various other sites are in direct correspondence with the numbered lines in the plots of $\langle \tilde{\mathbf{S}}_i \cdot \tilde{\mathbf{S}}_j \rangle$. Yellow windows indicate the Kitaev SL region.

To further confirm that a Kitaev spin liquid (SL) phase exists between the ferromagnetic (FM) and incommensurate (IC) ordered phases in our hyper-honeycomb system, we calculated the spin-spin correlation functions $\langle \tilde{\mathbf{S}}_i \cdot \tilde{\mathbf{S}}_j \rangle$ and compared them to those of the Kitaev SL phase of the 2D Kitaev-Heisenberg (KH) model on a honeycomb lattice[18]. The NN interactions of the KH model are written as

$$\mathcal{H}_{ij}^{(\gamma)} = 2K\, \tilde{S}_i^{\gamma} \tilde{S}_j^{\gamma} + J\, \tilde{\mathbf{S}}_i \cdot \tilde{\mathbf{S}}_j, \tag{12}$$

where $\gamma (= x, y, z)$ labels the three distinct types of NN bonds in the honeycomb plane. Following the notation of Ref. 18, we define the effective parameter $A = \sqrt{K^2 + J^2}$ and an angle φ via $K = A \sin\varphi$ and $J = A\cos\varphi$. In Fig. 4(a) the spin-spin correlations near the FM Kitaev limit ($\varphi = 1.5$) of the KH model are plotted, for a 24-site PBC cluster. The Kitaev SL state is characterized by a rapid decay of spin-spin correlations: in the Kitaev limit, only the NN correlations are finite and longer-range ones are zero; that is faithfully reproduced by the 24-site calculations. Even away from the Kitaev limit, the longer-range (not NN) spin-spin correlations fall within a narrow range $-0.05 \lesssim \langle \tilde{\mathbf{S}}_i \cdot \tilde{\mathbf{S}}_j \rangle \lesssim 0.1$ in the Kitaev SL phase ($1.40 < \varphi < 1.58$). As seen in Fig. 4(b), the hyper-honeycomb system exhibits similar features; the values of longer-range correlations are distributed within a narrow range $-0.03 \lesssim \langle \tilde{\mathbf{S}}_i \cdot \tilde{\mathbf{S}}_j \rangle \lesssim 0.1$ in the SL phase ($0.46 \lesssim J_2 < 1.58$). In other words, a rapid decay of the spin-spin correlations is seen in our hyper-honeycomb system, at the same level as in the Kitaev SL phase of the 2D KH model.

VI. ESTIMATION OF THE CRITICAL J_2 VALUES

In the main text we estimated the critical J_2 values for the SL-IC and IC-zigzag transitions by analyzing the propagation vector along the θ-direction in open clusters; the obtained values are $J_{2,c1} = 0.02$ and $J_{2,c2} = 1.43$ meV, respectively. The same analysis is possible for a propagation vector along the δ-direction, as shown in Fig. 5. We obtained very similar critical values, $J_{2,c1} = 0.02$, $J_{2,c2} = 1.48$ meV. This confirms the validity of our finite-size scaling analysis.

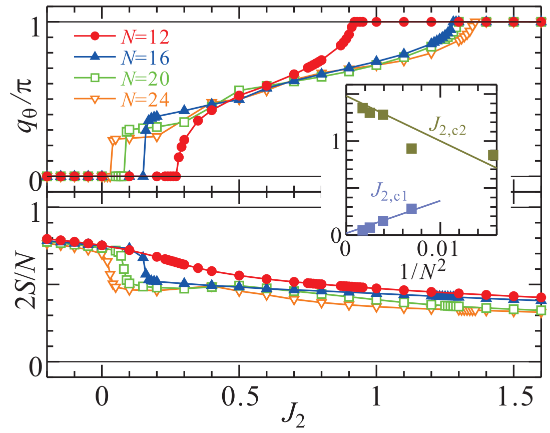

FIG. 5: Propagation vector q_θ^m and total spin $2S/N$ for our "open" clusters, as function of J_2. Inset: finite-scaling analysis of the critical points.

VII. SECOND DERIVATIVE OF THE GROUND-STATE ENERGY

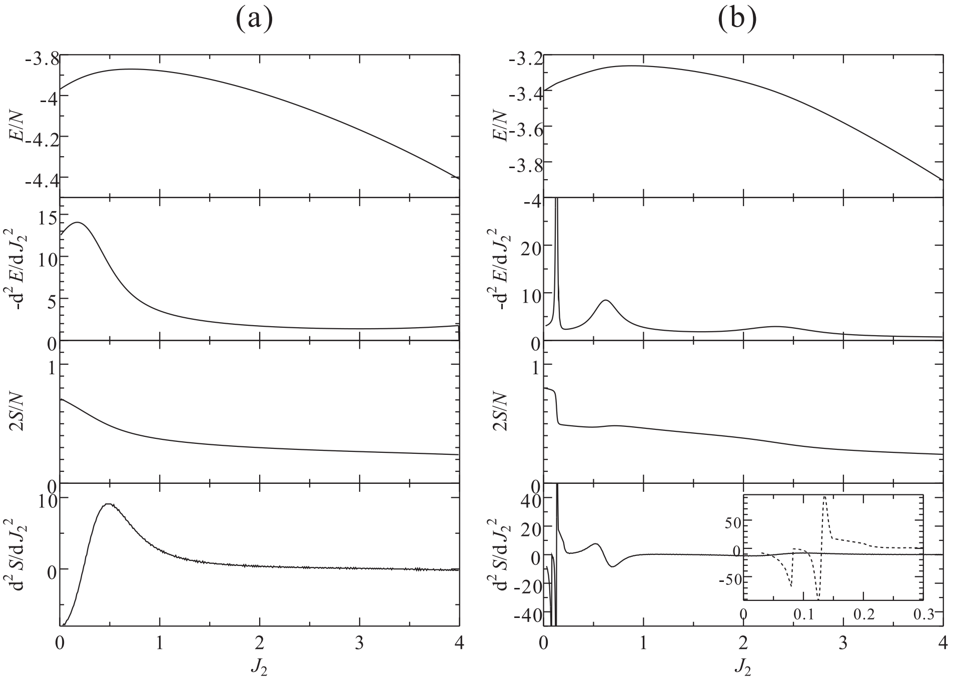

FIG. 6: Ground-state energy per site E/N and its second derivative $-d^2E/dJ_2^2$ plus total spin $2S/N$ and its second derivative $-d^2S/dJ_2^2$ for (a) the 24-site PBC and (b) the 16-site OBC clusters, as functions of J_2. Inset: enlarged view for the small-J_2 region.

As shown in Ref. 18, the second derivative of the ground-state energy E/N with respect to particular parameters may provide valuable information on the position of phase boundaries. In Fig. 6, we show results for $-d^2E/dJ_2^2$ for the 24-site PBC and 16-site OBC hyper-honeycomb clusters. The FM-SL phase boundary can be indeed identified by the peak position of $-d^2E/dJ_2^2$. We would like, however, to point out that this is not associated with a drastic change of the wave function.

The singularity of $-d^2E/dJ_2^2$ in the vicinity of the FM-SL transition is not really sharp even in the 2D KH model. Identifying the SL-to-IC and IC-to-zigzag-order transition points by $-d^2E/dJ_2^2$ is a rather delicate matter because those are continuous transitions and, for instance, the Bragg peak position in the spin structure factor changes continuously from 0 to π with increasing J_2. As seen in Fig. 6(b), $-d^2E/dJ_2^2$ exhibits two small peaks for J_2 values of about 0.65 and 2.35 meV. However, such peaks may also arise from finite-size effects for an incommensurate magnetic structure. The ground-state energy is lowered (raised) when the incommensurate propagation comes close to (gets away from) one of the discrete momenta possible for a small cluster.

As a result, the ground-state energy can heave up even when the propagation vector changes continuously. We also checked the second derivative of total spin, d^2S/dJ_2^2. Although that seems to provide less solid evidence of possible phase boundaries, the two singularities seen in the small-J_2 regime [bottom of Fig. 6(b)] do suggest FM-SL and SL-IC transitions.

[1] D. Figgen, K. A. Peterson, M. Dolg, and H. Stoll, J. Chem. Phys. **130**, 164108 (2009).
[2] T. H. Dunning, J. Chem. Phys. **90**, 1007 (1989).
[3] P. Fuentealba, H. Preuss, H. Stoll, and L. von Szentpaly, Chem. Phys. Lett. **89**, 418 (1982).
[4] V. M. Katukuri, H. Stoll, J. van den Brink, and L. Hozoi, Phys. Rev. B **85**, 220402 (2012).
[5] V. M. Katukuri, S. Nishimoto, V. Yushankhai, A. Stoyanova, H. Kandpal, S. K. Choi, R. Coldea, I. Rousochatzakis, L. Hozoi, and J. van den Brink, New J. Phys. **16**, 013056 (2014).
[6] N. A. Bogdanov, V. M. Katukuri, H. Stoll, J. van den Brink, and L. Hozoi, Phys. Rev. B **85**, 235147 (2012).
[7] H.-J. Werner, P. J. Knowles, G. Knizia, F. R. Manby, and M. Schütz, MOLPRO 2012, see http://www.molpro.net.
[8] K. Fink, R. Fink, and V. Staemmler, Inorg. Chem. **33**, 6219 (1994).
[9] A. B. van Oosten, R. Broer, and W. C. Nieuwpoort, Chem. Phys. Lett. **257**, 207 (1996).
[10] R. Broer, L. Hozoi, and W. C. Nieuwpoort, Mol. Phys. **101**, 233 (2003).
[11] C. J. Calzado, S. Evangelisti, and D. Maynau, J. Phys. Chem. A **107**, 7581 (2003).
[12] V. M. Katukuri, V. Yushankhai, L. Siurakshina, J. van den Brink, L. Hozoi, and I. Rousochatzakis, Phys. Rev. X **4**, 021051 (2014).
[13] N. A. Bogdanov, R. Maurice, I. Rousochatzakis, J. van den Brink, and L. Hozoi, Phys. Rev. Lett. **110**, 127206 (2013).
[14] J. Pipek and P. G. Mezey, J. Chem. Phys. **90**, 4916 (1989).
[15] T. Takayama, A. Kato, R. Dinnebier, J. Nuss, H. Kono, L. Veiga, G. Fabbris, D. Haskel, and H. Takagi, Phys. Rev. Lett. **114**, 077202 (2015).
[16] G. Jackeli and G. Khaliullin, Phys. Rev. Lett. **102**, 017205 (2009).
[17] T. Yildirim, A. B. Harris, A. Aharony, and O. Entin-Wohlman, Phys. Rev. B **52**, 10239 (1995).
[18] J. Chaloupka, G. Jackeli, and G. Khaliullin, Phys. Rev. Lett. **110**, 097204 (2013).
